# Commentary: Meditation Effects within the Hippocampal Complex Revealed by Voxel-Based Morphometric and Cytoarchitectonic Probabilistic Mapping

**DOI:** 10.3389/fpsyg.2015.01765

**Published:** 2015-11-19

**Authors:** Ayman Mukerji Househam, Zoran Josipovic

**Affiliations:** Psychology Department, New York UniversityNew York, NY, USA

**Keywords:** meditation, mindfulness, structural plasticity, hippocampus, subiculum, de-reification, non-conceptual awareness

The discovery of experience-dependent structural plasticity in the adult human brain is, arguably, one of the most significant recent developments in neuroscience (Maguire et al., 2000; Gage, 2002). The evidence of structural changes related to cognitive and motor practices, and skill acquisition, has inspired considerable clinical research into their potential relevance for diagnostic and therapeutic methods (Prosperini et al., 2015). Meditation and mindfulness include a wide variety of cognitive and affective practices, usually done long-term, with a broad impact that ranges from stress reduction and immune response enhancement, to optimizing cognitive functioning and affect regulation (Lutz et al., 2007). Since the first morphometric study of the effects of meditation by Lazar et al. (2005), a number of findings have been made showing increases in the volume and density of gray matter, and in the axonal connectivity of white matter (for review see Fox et al., 2014). An interesting finding has been that such changes could occur not only after many years of meditation practice, but also after a short 8-week training (Hölzel et al., 2011). Increases in volume or density of gray matter have been found in a number of areas involved in mediating meditation experience, such as the frontal pole, orbito-frontal cortex, anterior cingulate, insula, sensory cortices, and the hippocampal complex (Lazar et al., 2005; Hölzel et al., 2008; Luders et al., 2009). Increases in the axonal connectivity have been found in the corpus callosum and in the longitudinal fasciculus (Luders et al., 2011; Tang et al., 2012; Fox et al., 2014). Increases in gray matter volume have been found in both hippocampal and parahippocampal gyri (Hölzel et al., 2008; Leung et al., 2013), with some studies showing a larger right hippocampal gray matter increase (Hölzel et al., 2008; Luders et al., 2009), and others left (Hölzel et al., 2011; Luders et al., 2013). An ongoing issue for morphometric studies using MRI and PET has been disentangling what exactly is contributing to the observed increases in cortical thickness. As pointed out early on Lazar et al. (2005), an increase in the gray matter thickness could mean an increase in the number of neurons, but also an increase in dendritic arborization, glial cells, cerebral vasculature, or a combination of these factors.

The present study by Luders and colleagues is one of a number of studies in their excellent body of work on the effects of long-term meditation practice on the structural plasticity in the brain. It further extends the findings of previous studies, while resolving some of the methodological challenges inherent in this type of research. For this cross-sectional study, anatomical MRI scans were obtained from 50 meditators, ranging in experience from 4 to 46 years (average 20 years), and 50 matched non-meditator controls. After preprocessing, the whole brain voxel-wise analysis was performed, which revealed a cluster near the left hippocampus with significantly more gray matter in meditators than in controls, positively correlated with years of meditation practice. In order to more precisely explore the peri-hippocampal sub-regions that may have been involved, the study used cytoarchitectonic probabilistic mapping to define five such sub-regions in each hemisphere: cornu ammonis, entorhinal cortex, fascia dentata, hippocampal-amygdaloid transition area, and subiculum. All sub-regions, bi-laterally, had a significant main effect of group. The *post-hoc* comparison of regions showed significantly higher values for meditators in the right and the left subiculum, and a trend in the left hippocampal-amygdaloid transition area. All of the above regions were partially correlated with years of meditation practice, but only in the left subiculum was this statistically significant. Their subsequent analysis of the same data set found evidence for less age related gray matter atrophy in meditators than in controls (Kurth et al., 2015). While the cross sectional design of the study could not rule out the effect of selection bias, this latter finding suggests that the result was an effect of practice, in agreement with the findings of other morphometric studies that used short-term within-subject design (Hölzel et al., 2011). A subsequent study by the same authors found sex related differences in hippocampal volume, with greater increase in the left hippocampus for male meditators, and in the right hippocampus for female meditators (Luders et al., 2015). In discussing their findings the authors focus on the role of subiculum in stress regulation and, given a major effect of meditation in reducing stress, interpret them as most likely due to reduced stress-related atrophy.

Bearing the above in mind, an interesting question is what cognitive processes may have also contributed to the results. Although most meditation styles aim for a state of relaxed absorption, or awareness-presence that is free of conceptualization and goal-oriented activity (Josipovic, 2014), much of meditation, especially early on, is spent remembering instructions, monitoring how well one is performing them, and monitoring one's inner experience and the deviations from a remembered or imagined target state (Hasenkamp et al., 2012). Subiculum, which forms a major hippocampal output, is involved in episodic memory and spatial contextualization, as well as in comparing and integrating conflicting information (McNaughton, 2006; O'Mara et al., 2009). It receives inputs from the anterior cingulate cortex, involved in error detection, and from different attention networks via the hippocampus which receives inputs from all subcortical neurotransmitter systems involved in attention (Muzzio et al., 2009). Thus, the observed results of increased gray matter volume in subiculum could in part be due to different ways in which attention and memory converge during meditation. Future research could test whether increased subiculum volume is correlated with enhanced attention-memory efficiency. The authors suggest that during meditation the subiculum may be involved in recollecting experience based on previous experiences of meditation, in other word in constructing experience based on memory. Both retrospective and prospective memory can be engaged by meditation. Remembering how to practice within a meditation session, and how one should behave outside of it, forms a central part of mindfulness meditation (Vago and Silbersweig, 2012). Mindfulness meditation has also been found to increase the specificity of autobiographic memories (Crawley, 2015), which may be another factor contributing to the observed leftward asymmetry. However, in more advanced stages of meditation, the practitioner enters the unconstructed space of non-conceptual “direct” experience, in which one remains present in the immediacy of awareness free of mental constructions and elaborations (Reynolds, 1989). This aspect of meditation process is sometimes referred to as de-reification (Lutz et al., 2015). Future research could explore the depth of the non-conceptual states encountered in meditation. If, indeed, one can go beyond the mere cessation of subconscious inner speech, and experience a complete “absence of conceptually imputed existence of objects and persons,” in other words, have a fully conscious experience without any concepts, this could shed a new light on the nature of representation in the brain and on the role of the hippocampus in experience.

## Conflict of interest statement

The authors declare that the research was conducted in the absence of any commercial or financial relationships that could be construed as a potential conflict of interest.
